# Exploring the Intersection between Social Determinants of Health and Unmet Dental Care Needs Using Deep Learning

**DOI:** 10.3390/ijerph17197286

**Published:** 2020-10-06

**Authors:** Man Hung, Eric S. Hon, Bianca Ruiz-Negron, Evelyn Lauren, Ryan Moffat, Weicong Su, Julie Xu, Jungweon Park, David Prince, Joseph Cheever, Frank W. Licari

**Affiliations:** 1 College of Dental Medicine, Roseman University of Health Sciences, South Jordan, UT 84095, USA; rmoffat@roseman.edu (R.M.); jpark6@student.roseman.edu (J.P.); dprince@roseman.edu (D.P.); jcheever@roseman.edu (J.C.); flicari@roseman.edu (F.W.L.); 2 Department of Orthopaedic Surgery Operations, University of Utah, Salt Lake City, UT 84108, USA; bianca.ruiz.n@gmail.com; 3 Department of Economics, University of Chicago, Chicago, IL 60637, USA; ericstephenhon@uchicago.edu; 4 Department of Biostatistics, Boston University, Boston, MA 02115, USA; evelyn.lauren@utah.edu; 5 Department of Mathematics, University of Utah, Salt Lake City, UT 84112, USA; sochachai0880@gmail.com; 6 College of Nursing, University of Utah, Salt Lake City, UT 84112, USA; julie.xu@utah.edu

**Keywords:** unmet dental care need, artificial intelligence, deep learning, data science, machine learning, social determinants of health, precision dentistry, oral health outcomes

## Abstract

The goals of this study were to develop a risk prediction model in unmet dental care needs and to explore the intersection between social determinants of health and unmet dental care needs in the United States. Data from the 2016 Medical Expenditure Panel Survey were used for this study. A chi-squared test was used to examine the difference in social determinants of health between those with and without unmet dental needs. Machine learning was used to determine top predictors of unmet dental care needs and to build a risk prediction model to identify those with unmet dental care needs. Age was the most important predictor of unmet dental care needs. Other important predictors included income, family size, educational level, unmet medical needs, and emergency room visit charges. The risk prediction model of unmet dental care needs attained an accuracy of 82.6%, sensitivity of 77.8%, specificity of 87.4%, precision of 82.9%, and area under the curve of 0.918. Social determinants of health have a strong relationship with unmet dental care needs. The application of deep learning in artificial intelligence represents a significant innovation in dentistry and enables a major advancement in our understanding of unmet dental care needs on an individual level that has never been done before. This study presents promising findings and the results are expected to be useful in risk assessment of unmet dental care needs and can guide targeted intervention in the general population of the United States.

## 1. Introduction

Oral health plays a key role in the quality of life of an individual. Indeed, it is a gateway to one’s overall health and well-being [1,2,3]. Although societal oral health has been steadily improving, unmet dental care needs do remain. Unmet dental care needs are problematic and are a global public health concern. Previous research on unmet dental care needs has suggested that age, race, and socioeconomic factors are barriers of dental care [4,5]. In particular, social determinants of health are thought to be the root cause of unmet dental care needs and medical problems [6].

A retrospective study demonstrated that many states did not offer dental coverage for adults with Medicaid, forcing many people to resort to visiting the emergency departments for dental treatments [7]. In Rhode Island, the number of adults enrolled in the state Medicaid program increased after the implementation of the Affordable Care Act, yet the number of Medicaid enrollees who received dental care decreased [8]. It was concluded that this was due to the increasing number of private dental offices that stopped submitting Medicaid claims [8]. Additionally, many places that do not offer dental coverage for adults with Medicaid force patients to rely on emergency departments for dental treatments which are not equipped to provide preventive and specialized dental care [7]. Provider discontinuity [9], income level, normative treatment needs, and self-reported oral health status [5] are other characteristics that have been found to be associated with people with unmet dental needs as well. 

Research has been conducted to investigate the effect of dental insurance coverage on self-reported oral health. It found that those who were insured were more likely to be capable of performing all activities of daily living, and more likely to receive regular dental care [10]. This shows that lower dental cost due to the presence of dental insurance can lead to more regular dental visits and, consequently, less unmet dental needs.

Geographic barriers also play a role in perpetuating unmet needs and lower quality of care. Residents of rural areas in the United States have been found to be more likely to have unmet dental needs than city dwelling residents [11]. This correlates with the attitudes of the respective residents towards healthcare, and the distribution of dental professionals, with dentists favoring high income urban areas [12]. Currently, there are more than 4000 areas suffering from a shortage of providers. As such, many preventable diseases go untreated due to this lack of accessibility [13]. 

Although there is a preponderance of research studies examining the association between social determinants of health [14,15], there have been none so far utilizing a large national representative sample and a large array of factors to explore disparities of dental care needs. More specifically, there is a lack of studies employing a risk predictive model that can identify unmet dental care needs on an individual level using deep learning. Deep learning [4], a tool in artificial intelligence, has already revolutionized the areas of business, robotics, gaming, and computation. It is currently making rapid strides in medical diagnosis, molecular activity determination, genomic analysis, and image and sound recognition. It represents an emerging and promising analytical approach in dentistry. 

In this study, we were interested in exploring what affects the quality of healthcare, particularly as it relates to unmet dental care needs. The purpose of this study was threefold: (1) To examine the difference of social determinants of health between those who had unmet dental care needs and those who did not have unmet dental care needs, (2) to identify factors predictive of unmet dental care needs, and (3) to develop a risk prediction model to predict unmet dental care needs on an individual level using deep learning. This study can aid dentists and health care professionals to identify high-risk individuals for appropriate courses of dental treatment. It can also provide more insight into health care disparities and social determinants of health, and the effects and effectiveness of government healthcare reform.

## 2. Materials and Methods 

### 2.1. Study Design

We used the 2016 Medical Expenditure Panel Survey (MEPS) [16] data for conducting this study. MEPS is a nationally representative survey, sponsored by the Agency for Healthcare Research and Quality, which measures access, use and cost of healthcare services. MEPS is the most complete source of data on the cost and use of health care and health insurance coverage. The survey consists of three major components, namely (1) household, (2) medical provider, and (3) health insurance. The household component (HC) samples are drawn from the respondents to the National Health Interview Survey by the National Center for Health Statistics [16]. The medical provider component covers hospitals, physicians, home health care providers, and pharmacies identified by MEPS-HC respondents. The health insurance component contains data from a sample of private and public sector employers on health insurance plans they offer their employees, also known as the Health Insurance Cost Study. This study utilized data from the MEPS-HC and included samples of all ages, who had no missing data for these two variables indicative of the outcome “unmet dental care needs”: (1) Unable to get necessary dental care (yes/no); (2) delayed in getting necessary dental care (yes/no). For those who were under the age of 18 years old, a household adult member responded to the MEPS-HC’s survey questions for the minor.

### 2.2. Outcome

The target variable was unmet dental care needs, with a binary outcome of either yes or no. This variable was created using two self-reported questions from participants at the end of the year 2016: (1) Unable to get necessary dental care, (2) delayed in getting necessary dental care. If a participant responded “yes” to at least one of these two self-reported questions, they were considered as having unmet dental care needs. Otherwise, they did not have unmet dental care needs. 

### 2.3. Predictors

The 2016 MEPS dataset consisted of 1941 variables. We excluded the outcome variable and all of the variables (e.g., replicated variables that have different scales or measured at different survey rounds, IDs, weights, respondent units, imputation flags) that were not useful as predictors for unmet dental care needs. Based on expert knowledge and team consensus, a total of 237 relevant variables were selected and kept as potential predictors to predict unmet dental care needs. These 237 predictors included categories of demographic characteristics, physical health status, mental health status, socioeconomic indicators, employment, insurance coverage, healthcare providers, visits, and charges. They were measured at the beginning of the year 2016. 

### 2.4. Analyses

We intentionally used the measurement at the beginning of the year for the predictors and measurement at the end of the year for the outcome in order to establish temporal precedence for causal relationship inference. Descriptive statistics were computed to examine sample characteristics using mean, standard deviation, count and proportion as appropriate. Sampling weights were applied to obtain prevalence estimates that were representative of the United States population. 

To prepare the data for pre-processing, responses containing negative values were assigned as not applicable, which were considered as missing in data analyses. Afterward, variables containing more than 20% missing were dropped. Non-predictor variables such as data collection variables (e.g., responding unit, imputation flags) as well as respondent IDs were excluded. Redundant variables (e.g., income as both categorical and continuous, multiple variables to indicate race and ethnicity) were also excluded. Since responses were collected multiple rounds throughout the year, only variables collected on the earliest round were included for the predictors, and on the latest round for the outcome. Specific variables (e.g., total amount paid by Medicare, total amount paid by Medicaid) adding up to a general variable (e.g., total healthcare expenditure) were removed to eliminate redundancy.

The outcome variable contained highly unbalanced classes of data. It is highly skewed towards individuals with met dental needs. In order to prepare the data to input into the machine learning models, cases with unmet dental need were replicated using oversampling to achieve balance. One-hot-encoding was applied towards the categorical variables to turn them into binary variables. It is a common technique to covert categorical data into numerical form to enable efficient implementation of machine learning algorithms. All data were normalized so that the predictor variables were on the same scale. The data were then split into 60% training and 40% test sets.

#### 2.4.1. Social Determinants of Health

The differences of social determinants of health between those who had unmet dental care needs and those who did not have unmet dental care needs were examined using chi-square tests for the categorical variables. 

#### 2.4.2. Top Predictors of Unmet Dental Care Needs

A machine learning method [17,18,19] called decision tree classifier [20,21] was used to determine the top features (i.e., variables) that were predictive of unmet dental care needs. There are two general types of decision trees in the Scikit package utilized in this study: classification and regression trees (CART). [22] Under the general umbrella of CART, classification is suitable for use in discrete outcome variable and regression is appropriate for continuous outcome variable. Since the outcome variable for this study was discrete in nature (e.g., had unmet dental care needs versus did not have unmet dental care needs), DecisionTreeClassifier (e.g., classification) in Scikit [23,24] was used with a maximum depth of 8, maximum leaf node of 300, minimum sample split of 100, and minimum samples leaf of 20. Relative variable importance scores were computed for the top predictors. The decision tree classifier was chosen due to its interpretability and flexibility for simultaneous inclusion of both categorical and numerical values and its ability to sum feature importance of a set of features and normalize them.

#### 2.4.3. Model for Risk Predictor of Unmet Dental Care Needs

A machine learning method called deep learning [25,26,27] was used to generate a model for risk prediction of unmet dental care needs for individuals. Deep learning is a major breakthrough in the field of artificial intelligence that has the ability to perform exceptionally well with large and complex data. It was chosen over traditional machine learning methods (such as support vector machine, k-nearest neighbors, etc.) in this study because traditional methods were suitable for simpler data and more straightforward feature engineering, but deep learning was more appropriate for complex feature engineering such as images and videos and/or large amount of data like what we had in our study. Additionally, deep learning has the ability to produce highly accurate model in the face of complex data where traditional methods fall short. Since model accuracy was regarded as the key performance indicator in this study, deep learning was the method of choice.

The deep learning model had three middle layers with 100 nodes in each layer. Bayesian optimization was applied to deep learning to fine tune the model. Sklearn Pipeline and Keras Wrappers were combined for fine tuning hyperparameters that were arranged in five sequential blocks with one fully connected hidden layer of 100 nodes and rectified linear unit activation for learning initialization followed by a fully connected output layer with 10 nodes of rectified linear unit activation, in addition to the softmax layer of 2 nodes at the end of the model. Several key performance indicators such as sensitivity, specificity, accuracy, precision, and area under the curve (AUC) were computed. 

Analyses in this study were performed using R (R Foundation for Statistical Computing, Vienna, Austria) [28] for traditional statistics and using Python (Python Software Foundation, Beaverton, OR, USA) [29] for decision tree classifier and deep learning. Statistical tests with *p* < 0.05 two-sided were considered as significant.

## 3. Results

There was a total of 33,929 participants included in the study, representative of 323,141,687 of the United States population, with an average age of 46.5 years (standard deviation = 18.0 years). There were 52.3% female and 42.0% white. More than half of the population reported having private health insurance coverage, but only one third had dental insurance. See Table 1 for more information.

Table 2 displays social determinants of health variables and results related to the differences of these social determinants of health by unmet dental care needs. There were more people who did not have dental insurance (5.6%) than those who had dental insurance (3.6%) that reported unmet dental care needs. There were relatively higher proportions of individuals who had public health insurance that had unmet dental care needs (5.9%) than those who had private health insurance (4.1%). Approximately 6.2% of those who were 65 years or older experienced unmet dental care needs, but only 4.7% of those under 65 years old did.

Unmet medical care needs were notably related to unmet dental care needs. Of those who had unmet dental care needs, 28.4% were delayed in getting necessary medical care, 37.6% were unable to get necessary medical care, and 31.9% were unable to get necessary prescription medication. There were 15.7% of those who had poor mental health status having unmet dental care needs while only 3.2% of those who had excellent mental health status had unmet dental care needs (Table 3).

**Figure 1:**
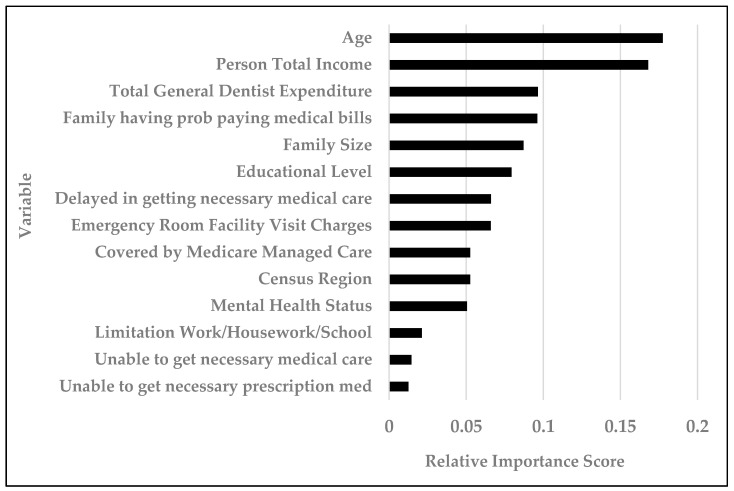
Relative importance of variables in predicting unmet dental care needs.

Among the 33,929 records and 237 variables entered in the prediction of unmet dental care needs, the decision tree classifier identified fourteen important variables: (1) Age, (2) personal total income, (3) total general dentist expenditure, (4) family having problems paying medical bills, (5) family size, (6) educational level, (7) delayed in getting necessary medical care, (8) emergency room facility visit charges, (9) covered by Medicare managed care, (10) census region of residence, (11) mental health status, (12) limitation at work/house/school, (13) unable to get necessary medical care, and (14) unable to get necessary prescription medication (Table 3 and Figure 1). In other words, these fourteen variables had the most impact on determining whether an individual had unmet dental care needs or not. Figure 1 displays the relative variable importance score of all these fourteen variables, with age and person total income as the top two most important variables. In constructing a prediction model, a variable is considered as important when the exclusion of this variable causes the prediction model’s error to increase, because the model relied heavily on this variable for the prediction. The most important variable is the variable that results in the greatest model error when the variable is taken away from the model. 

**Figure 2:**
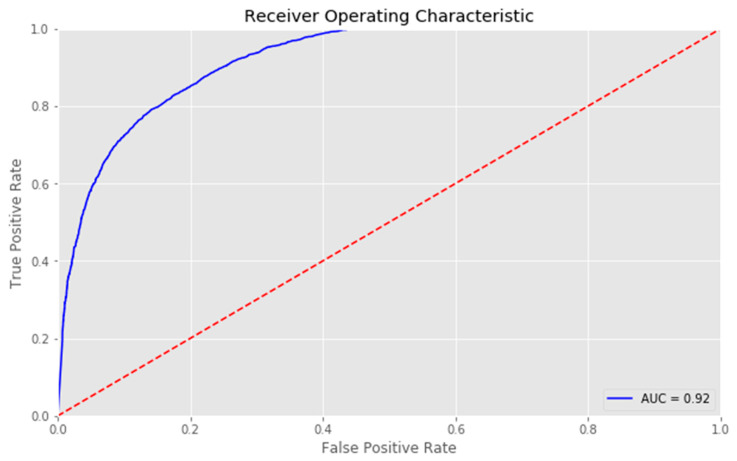
Receiver operating characteristic curve in risk prediction of unmet dental care needs using deep learning.

The prediction model for unmet dental care needs using deep learning performed very well. It reached an accuracy of 82.6%, sensitivity of 77.8%, specificity of 87.4%, precision of 82.9%, and an AUC of 0.918 (Figure 2).

## 4. Discussion

Unmet dental care needs are a significant public health concern in the United States. Exploring the intersection between the social determinants of health and unmet dental care needs and developing a risk prediction model presents an opportunity to more easily identify patients who are at risk of not receiving necessary care. The use of deep learning in artificial intelligence to develop a risk prediction model is a significant step forward in improving oral health. 

This study demonstrated that social determinants of health are strong risk predictors of unmet dental care needs. Unsurprisingly, unmet dental care needs were found more often in those who did not have dental insurance than those who had dental insurance. But an unexpected finding was that those with no health insurance and those with private health insurance all had a lower level of unmet dental care needs than those with public dental insurance. This is probably due to the fact that the number of Medicaid enrollees has increased while the number of dental providers participating in Medicaid program has concurrently decreased due to their dissatisfaction with the Medicaid program [8]. Thus, there is a lower capacity of dental providers meeting the dental needs for those who have Medicaid or public dental insurance in general. The Medicaid program also has the stress of the increase cost of dental expenditures that surpasses the average inflation rate. In 2016, the total US dental expenditures were above $124 billion when compared to around $50 billion in 1995, a 150% increase. The projected US dental expenditures will reach $192 billion by 2026, which is another 50% increase from the 2016 expenditure. The higher annual increase rate will only cause more pressure on those individuals with unmet dental care.

Our findings also support research conducted by Ku et al. (2008) that uninsured adults appeared to be somewhat healthier than Medicaid insured recipients [30]. This raises a critical question as to why the United States is relying on a Medicaid program that is failing to meet the needs or protecting the health of the public. Perhaps it is time to consider quality care for all, instead of Medicaid or Medicare for all. 

This study also found that females were more likely to have unmet needs than males. Previous research has suggested that this is due to economic reasons [5,31], and without our society eradicating gender inequality in the workforce, this can be a discernible trend. Race/ethnicity was also a significant discriminator of unmet dental care needs, with those reporting other or multiple race/ethnicity had the highest rate of unmet needs at 10.9%. Past studies have suggested that racial/ethnic dental health disparities are mainly due to socioeconomic factors [32,33,34]. Understanding racial/ethnic disparities will help dental providers to bridge unexplained gaps in our society and suggest strategies for interventions and public health reformations population-wide. 

Of the 237 variables explored in this study, 14 of them were found to be highly predictive of unmet dental care needs. Figure 1 displays the relative importance of each of the 14 predictors, with age as the most important predictor. A previous study suggests that older edentulous patients visit the dentist because they have to, rather than for preventive reasons [35]. With many individuals of the Baby Boomer generation retiring and relying on public health insurance for dental care needs, age and socioeconomic status becomes highly relevant in determining whether or not dental care needs are being met. Naturally, a person’s total income was found to be the second most important predictor. This confirms what has been shown by Edelstein et al. that children living in poverty consistently suffer from more tooth decay and have more unmet dental care needs than do their more affluent peers [14]. Additionally, research conducted by Chae et al. confirmed that the socioeconomically vulnerable elder population were more likely to experience high levels of unmet dental care needs [36]. Both the geriatric population and populations living in less affluent socioeconomic conditions are more likely to have public dental insurance such as Medicaid, which contributes to greater unmet dental care. 

In this study, the use of machine learning methods such as deep learning helped us to develop precise computer algorithms to model the risk of unmet dental care needs for the United States population. Indeed, machine learning helped identify social determinants and risk predictors for unmet dental care needs, but more importantly it helped us generate algorithms that are able to consider combinations of variables to assist the dental practitioner in risk assessment in clinical scenarios for the future. Algorithms developed from machine learning are the driving force behind artificial intelligence as experienced in self-driving cars, facial identification for unlocking phones, space shuttle, and other robotics used in our lives. Machine learning has powerful dental public health implications as it can disrupt and advance areas of diagnosis and treatment in oral health. The machine learning algorithms can be used in diagnosis in dental practices or in online modules such as teledentistry to provide recommendations for dental examinations and treatment for those identified as high risk. They may also be used by non-dental professionals to categorize as high-risk those patients that have limited access to care, or as seen in our study, are elderly or have limited financial resources. Machine learning may enable the development of cost-efficient practices in dentistry and has huge implications for the future of more comprehensive care for individuals.

However, this study was not without limitations. The data collected from MEPS may not represent the changing population of the United States. Although the data were collected in the year of 2016, a rapid change in the population dynamics may contribute to changing patient demographics and other characteristics over time. Onsite clinical validation in the future may be needed to further improve the algorithms. 

## 5. Conclusions

Unmet dental healthcare needs have a longstanding history in the United States and show no signs of abatement. Understanding its social determinants allows us to tackle unmet needs with more focused intensity and better allocation of resources. Innovation using artificial intelligence such as machine learning is a great way to tackle this age-old problem, which enables the development of more precise and effective diagnostic modalities for dental practitioners. The model developed in this study will enable early identification and concentration upon those who are most at-risk for not receiving dental treatment. On a larger scale, these machine learning algorithms may create more automated and financially feasible systems of dental healthcare delivery on a national level.

## Figures and Tables

**Table 1:** Demographic characteristics.

Variable	Description	*N* *	% *	Mean	Standard Deviation *	Median *
AGE16X	Age (year)	25,200 (246,354,311)		46.5 (47.5)	18.0 (18.2)	45 (47)
TTLP16X	Person’s total income ($)	22,171 (209,529,021)		31,402 (38,972)	20,800 (41,802)	20,800 (28,000)
RACETHX	Race/Ethnicity					
	Hispanic	7273 (58,128,006)	29.2 (18.0)			
	White	10,467 (194,556,659)	42.0 (60.2)			
	Black	4536 (39,595,626)	18.2 (12.3)			
	Asian	1923 (18,459,241)	7.7 (5.7)			
	Other race or multiple race	706 (12,402,154)	2.8 (3.8)			
INSCOV16	Insurance coverage					
	Private	18,553 (216,879,523)	53.5 (67.1)			
	Public	12,255 (81,653,479)	35.4 (25.3)			
	Uninsured	3.847 (24,608,684)	11.1 (7.6)			
HWELLSPE	How well person speaks English					
	Very well	7010 (46,178,975)	58.0 (65.1)			
	Well	1929 (10,566,967)	16.0 (14.9)			
	Not well	2013 (9,281,478)	16.6 (13.1)			
	Not at all	1139 (4,900,558)	9.4 (6.9)			
BORNUSA	Person born in US					
	Yes	27,040 (276,843,356)	78.4 (85.9)			
	No	7471 (45,524,308)	21.6 (14.1)			
DNTINS16	Have dental insurance					
	Yes	11,834 (139,923,837)	34.4 (43.7)			
	No	22,565 (180,455,575)	65.6 (56.3)			
LANGSPK	Language spoken at home other than English					
	Spanish	9947 (49,213,364)	74.0 (62.6)			
	Another language	3497 (29,375,518)	26.0 (37.4)			
SEX	Sex					
	Male	16,526 (158,186,085)	47.7 (49.0)			
	Female	18,129 (164,495,602)	52.3 (51.0)			
MARRY16X	Marital status					
	Married	12,139 (130,618,832)	35.0 (40.4)			
	Widowed	1607 (15,549,509)	4.6 (4.8)			
	Divorced	2945 (27,603,935)	8.5 (8.5)			
	Separated	768 (5,247,036)	2.2 (1.6)			
	Never married	9085 (79,512,517)	26.2 (24.6)			
	Under 16—Not applicable	8102 (64,609,857)	23.4 (20.0)			
HIDEG	Highest degree					
	No degree	5515 (36,618,014)	16.1 (11.4)			
	GED	1095 (9,796,007)	3.2 (3.0)			
	High school diploma	10,805 (107,047,499)	31.5 (33.3)			
	Bachelor’s degree	3946 (48,164,057)	11.5 (15.0)			
	Master’s degree	1759 (21,623,234)	5.1 (6.7)			
	Doctorate degree	425 (5,624,308)	1.2 (1.7)			
	Other degree	1961 (21,716,535)	5.7 (6.8)			
	Under 16—Not applicable	8813 (70,802,893)	25.7 (22.0)			

* Values inside the parentheses are weighted prevalence.

**Table 2:** Social determinants of health by unmet dental care need.

Variable	Description	Have Unmet Dental Need		*p*-Value	95% CI of *p*-Value
		Yes	No		
DNTINS16	Dental insurance	*n* (%)	*n* (%)	<0.001	0.000–0.000
	Yes	421 (3.6)	11,310 (96.4)		
	No	1248 (5.6)	20,892 (94.4)		
INSCOV16	Health insurance coverage	*n* (%)	*n* (%)	<0.001	0.000–0.000
	Private	754 (4.1)	17,565 (95.9)		
	Public	702 (5.9)	11,234 (94.1)		
	Uninsured	213 (5.8)	3461 (94.2)		
AGE16X	Age	*n* (%)	*n* (%)	<0.001	<0.001
	Under 65 years	1385 (4.7)	27,911 (95.3)		
	65 years and over	284 (6.2)	4291 (93.8)		
SEX	Sex	*n* (%)	*n* (%)	<0.001	<0.001
	Male	687 (4.3)	15,476 (95.7)		
	Female	982 (5.5)	16,784 (94.5)		
RACETHX	Race/Ethnicity	*n* (%)	*n* (%)	<0.001	0.000–0.000
	Hispanic	425 (3.8)	10,635 (96.2)		
	White	696 (5.4)	12,245 (94.6)		
	Black	356 (5.7)	5904 (94.3)		
	Asian	92 (3.8)	2357 (96.2)		
	Other race or multiple race	100 (8.2)	1119 (91.8)		
BORNUSA	Person born in the US	*n* (%)	*n* (%)	0.745	0.745
	Yes	1310 (6.3)	25,156 (93.7)		
	No	358 (5.0)	7013 (95.0)		
HWELLSPE	How well person speaks English	*n* (%)	*n* (%)	0.146	0.140–0.154
	Very well	292 (4.2)	6637 (95.8)		
	Well	99 (5.2)	1806 (94.8)		
	Not well	101 (5.1)	1890 (94.9)		
	Not at all	46 (4.1)	1074 (95.9)		

**Table 3:** Top predictors of unmet dental care needs.

Variable	Description	Have Unmet Dental Need		*p*-Value	95% CI of *p*-Value
		Yes	No		
MDDLAY42	Delayed in getting necessary medical care	*n* (%)	*n* (%)	<0.001	0.000–0.000
	Yes	277 (28.4)	698 (71.6)		
	No	1391 (4.2)	31,533 (95.8)		
PROBPY42	Family having problems paying medical bills	*n* (%)	*n* (%)	<0.001	0.000–0.000
	Yes	546 (14.7)	3177 (85.3)		
	No	1111 (3.7)	29,030 (96.3)		
MDUNAB42	Unable to get necessary medical care	*n* (%)	*n* (%)	<0.001	0.000–0.000
	Yes	225 (37.6)	373 (62.4)		
	No	1443 (4.3)	31,849 (95.7)		
PMUNAB42	Unable to get necessary prescription med	*n* (%)	*n* (%)	<0.001	<0.001
	Yes	170 (31.9)	363 (68.1)		
	No	1495 (4.5)	31,847 (95.5)		
ACTLIM31	Limitation work/Housework/School	*n* (%)	*n* (%)	<0.001	<0.001
	Yes	373 (13.9)	2312 (86.1)		
	No	1251 (4.4)	27,085 (95.6)		
REGION31	Census region	*n* (%)	*n* (%)	0.009	0.008–0.012
	Northeast	220 (4.2)	5066 (95.8)		
	Midwest	304 (4.8)	6050 (95.2)		
	South	651 (5.1)	12,067 (94.9)		
	West	485 (5.4)	8524 (94.6)		
MNHLTH31	Mental health status	*n* (%)	*n* (%)	<0.001	0.000–0.000
	Excellent	483 (3.2)	14,458 (96.8)		
	Very Good	392 (4.6)	8046 (95.4)		
	Good	498 (6.5)	7143 (93.5)		
	Fair	217 (11.7)	1633 (88.3)		
	Poor	69 (15.7)	371 (84.3)		
MCRPHO31	Covered by Medicare managed care	*n* (%)	*n* (%)	<0.001	0.000–0.000
	Coverage by Medicare managed care	148 (8.9)	1517 (91.1)		
	Coverage by Medicare—not managed care	220 (7.5)	2699 (92.5)		
	Not covered by Medicare	1261 (4.5)	27,040 (95.5)		
